# Plants, Pollinators and Pheromones: Promises and Lies of Semiochemicals

**DOI:** 10.1111/pce.15670

**Published:** 2025-06-04

**Authors:** Filip Slavković, Abdelhafid Bendahmane

**Affiliations:** ^1^ Institute of Plant Sciences Paris‐Saclay (IPS2), INRAE, CNRS University of Paris‐Saclay, University of Evry, University of Paris Cité Gif‐sur‐Yvette France

**Keywords:** deceptive pollination, pheromones, pollinators, secondary metabolites, semiochemicals, VOCs

## Abstract

Pollination is traditionally regarded as a quintessential mutualism, yet many plants employ deceptive strategies to achieve reproductive success. Among the most intriguing is sexual deception, wherein flowers mimic the sex pheromones and visual signals of female insects to attract male pollinators—without providing any reward. This strategy, most notably observed in orchids, is a powerful driver of diversification and speciation. Recent advances in genomics, metabolomics, and high‐resolution imaging are shedding light on the genetic and biochemical mechanisms underpinning these complex mimicry systems. Remarkably, subtle genetic modifications and the repurposing of existing gene networks can give rise to highly specialized and effective forms of deception. Central to this process are volatile organic compounds (VOCs), which serve as species‐specific semiochemicals that manipulate innate pollinator behaviors and reinforce reproductive isolation. This review synthesizes emerging insights into floral semiochemistry and highlights its broader applications in pollinator surveillance, crop pollination enhancement, and biodiversity monitoring. As global pollinator populations face increasing threats, understanding floral chemical ecology offers promising avenues for designing pollinator‐friendly crops and advancing tools in synthetic ecology.

## Summary statement

Flowering plants employ a mix of chemical, visual, and structural mimicry to attract pollinators. This review highlights recent findings on plant‐insect communication and explores their potential applications in pollinator management and conservation.

## 1 Plants, Pollinators, and Deception

Coevolution has long been regarded as one of the major processes creating biodiversity (Ehrlich and Raven 1964; Suchan and Alvarez 2015). For at least the last 100 million years (Cardinal and Danforth 2013), pollinators have coevolved with plants, ensuring the collection of food, while securing plant reproduction and fitness. Yet, what happens when this mutualistic interaction is not respected by one of the partners? A paradigmatic example of deceptive pollination which includes food or sexual deception (Jersáková et al. 2006). Sexual deception is a pollination strategy in which flowers mimic the sex pheromones and appearance of female insects to attract male insect pollinators. Cunningly, the deceptive plants use sophisticated complex traits with a combination of olfactory, visual, and morphological mimicry (De Jager and Peakall 2016; Wong et al. 2022; Perkins et al. 2023; Phillips et al. 2024), with floral odor signals playing major roles in these interactions (Schiestl et al. 2004; Phillips and Peakall 2018). As a result, often, but not necessarily, pollination occurs during attempted copulation with the flower (Ayasse et al. 2003; Bohman et al. 2016). Among pollinators, numerous insect groups, such as male bees and wasps (Bohman et al. 2016; Peakall et al. 2020), fungus gnats, and beetles, among others (Cohen et al. 2021; Hayashi et al. 2021), are deceived by this trickery.

Insect pollinators are crucial for the productivity of agricultural systems, with 75% of crop species and 35% of global crop production being dependent on insect pollinators (Klein et al. 2007; Powney et al. 2019; Slavković et al. 2021). From the economic perspective, the pollination services provided by insect pollinators have an estimated value of US$29 billion in the USA (Calderone 2012) and range widely from US$195 billion to ~US$387 (US$267–657) billion per year worldwide (Porto et al. 2020). Among commercially important crops that employ deceptive pollination strategies is *Vanilla sp*. (Pansarin 2022), the fruits of which are almost exclusively produced by hand pollination, since, under natural conditions, the fruit set is low due to the scarcity of pollinators (Soto‐Arenas 1999). Having in mind pollinator decline in different regions (Goulson et al. 2015; Powney et al. 2019; Gray et al. 2019), studying plant‐pollinator interactions is critical and timely (European Commission, Directorate‐General for Environment, Pollinators – Importance for nature and human well‐being, drivers of decline and the need for monitoring 2020; Slavković and Bendahmane 2023). In this review, we summarize recent advances in semiochemicals (SCs) and floral mimicry used in deceptive pollination *sensu lato*, discuss their applications in agriculture and conservation studies and suggest future research directions.

## 2 Research on SCs: Then and Now

Research about the role of floral volatile organic compounds (VOCs) in the pollination strategy of sexual deception has almost a century long history (Bohman et al. 2020a). One of the earliest reports was documented in 1927 by Edith Coleman who found that Australian *Cryptostylis* orchids were exclusively pollinated by the males of an ichneumonid wasps (Peakall 2023). Several decades later, in 1948, Kullenberg started a multidecade study of an orchid *Ophrys* (Kullenberg 1961). Impressively, in Orchidaceae, about one‐third of the estimated 18500 species are thought to be pollinated by deceit (Cozzolino and Widmer 2005) suggesting an important role of floral deception and specialization in species diversification (Cozzolino and Widmer 2005; Ayasse et al. 2011). In the last 20 years, a remarkable progress has been made on the subject and the research continues to grow (Perkins et al. 2023). First confirmed reports of sexual deception outside the Orchidaceae have recently been described in ornamental crops of Asteraceae (Ellis and Johnson 2010) and Iridaceae (Vereecken et al. 2012) showing that pollination by deceit is not foreign to other plant families. Until today, pollination by sexual deception has been reported in various parts of the world, with four centers of sexually deceptive plant diversity including Oceania, Europe, Africa and South America (Peakall 2023).

**Figure 1:**
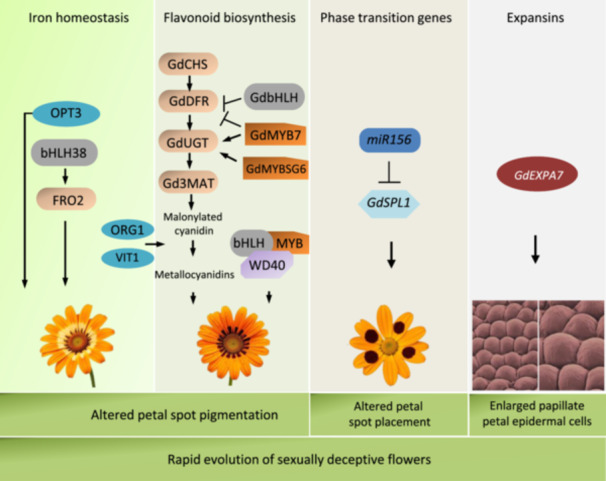
Genetic control of mimicry in sexually deceptive flowers. Model adapted from Kellenberger et al. 2023 on *Gorteria diffusa* which includes co‐option of genes involved in iron homeostasis, flavonoids biosynthesis, phase transition and cell structure genes. Iron homeostasis genes associated with petal spot pigmentation include *OBP3‐RESPONSIVE GENE ORG1*; *ORG2* (bHLH038) which induces the expression of many iron uptake genes, including *FERRIC REDUCTION OXIDASE 2* (*FRO2*); *OLIGOPEPTIDE TRANSPORTER 3* (*OPT3*) and *VACUOLAR IRON TRANSPORTER 1* (*VIT1*). Genes involved in anthocyanin and flavonoid biosynthesis pathways reported in *Gorteria diffusa* are *CHALCONE SYNTHASE* (*GdCHS*), *DIHYDROFLAVONOL 4‐REDUCTASE* (*GdDFR*), *ANTHOCYANIDIN SYNTHASE* (*GdANS*), *UDP‐GLYCOSLYTRANSFERASE* (*GdUGT*), *ANTHOCYANIN 3‐O‐GLUCOSIDE‐6″‐O‐MALONYLTRANSFERASE* (*Gd3MAT*). In addition, GdbHLH, GdMYB7, and GdMYBSG6 are homologous to characterized TFs from other species, which form the anthocyanin‐regulating MYB‐bHLH‐WD40 TF complexes. The miR156‐GdSPL1 transcription factor module controls petal spot placement, while expansin gene EXPA7 positively regulates the formation of enlarged papillate petal epidermal cells.

Today, the availability of state‐of‐the‐art ‐omics technologies (e.g. genomics, transcriptomics, proteomics, metabolomics) sheds new light on our understanding of the biosynthesis of specialized metabolites involved in plant‐pollinator interaction. In addition, high‐precision imaging facilities such as microcomputed tomography allow for detailed three‐dimensional phenotypic analyses of floral morphologies in the context of plant‐pollinator interactions (Begot et al. 2022). Recently, the molecular basis of several sexually deceptive flowers has been described (Sedeek et al. 2013; Xu et al. 2017). In addition, recent studies have unraveled the genetic basis of species‐specific differences in floral scent variation in sexually deceptive *Ophrys*. Namely, two specific stearoyl–acyl carrier protein desaturases, SAD2 and SAD5, were identified as key contributors to reproductive isolation (Xu and Schlüter 2015), while amino acid change in SAD5 enabled mimicry of the pollinating bee *Colletes cunicularius* sex pheromone (Sedeek et al. 2016). Similarly, specific SAD homologs caused a difference in alkene double‐bond positions responsible for reproductive isolation between *O. exaltata* and closely related species *O. sphegodes* (Xu et al. 2012). These data showed that even minor mutations in the underlying genetic networks are sufficient to drastically affect mimicry (Xu and Schlüter 2015; Sedeek et al. 2016; Kellenberger et al. 2023), which corroborates that minor changes in floral odor bouquets are prerequisites for pollinator shifts and speciation events (Vereecken et al. 2010). This year (2024), the genome of the early spider‐orchid *Ophrys sphegodes* was published, providing evidence that gene duplication contributed to the evolution of chemical mimicry. Moreover, the authors reported a highly differentiated genomic candidate region for pollinator‐mediated evolution (Russo et al. 2024). The evolution of sexually deceptive flowers requires orchestrated changes in several genetic networks altering multiple unrelated floral features, which classifies sexual deception as a composite novelty (Kellenberger et al. 2023). The genetic network controlling mimicry in sexually deceptive South African beetle daisy (Asteraceae) is shown in Figure 1. Strikingly, this recent publication suggested that the integration of multiple co‐opted genetic elements facilitated the rapid evolution of complex petal spots that mimic female bee‐fly pollinator (Kellenberger et al. 2023). First, co‐option of iron homeostasis genes is associated with altered petal spot pigmentation, similar to that of female pollinators, which requires antocyanin‐regulating MYB‐bHLH‐WD40 TF complexes (Yan et al. 2021). Second, co‐option of the root hair gene *GdEXPA7* elicited copulation responses from male flies, and third, co‐option of the miR156‐GdSPL1 transcription factor module altered petal spot placement, resulting in better mimicry of female flies. These findings suggest that, in sexually deceptive flowers, a modular integration of multiple independently co‐opted genes can speed up the evolution of complex phenotypic novelties (Kellenberger et al. 2023).

When it comes to floral volatiles, current theory predicts that chemical communication can arise from compounds primarily evolved for noncommunicative purposes, as insect pheromones originated from extant precursor compounds being selected for information transfer (Stökl and Steiger 2017). In floral mimicry, a key gene for signal production has evolved by gene duplication from a housekeeping gene involved in fatty acid metabolism (Schlüter et al. 2011). Similarly, many volatiles, pigments, and even some rewards are thought to have shifted from a primary defense function to the attraction of pollinators (Borghi et al. 2017).

## 3 Chemistry of Deception: The Nature of Inviting

The chemical structure of insect pheromones is widely diverse (Rizvi et al. 2021) and therefore it is not surprising that SCs used in mimicry come in a variety of forms. According to their chemistry and biosynthesis, floral volatiles have been classified into a few basic categories, namely: fatty acid derivatives, terpenoids, benzenoids/phenylpropanoids and N‐ and S‐ bearing compounds (Vivaldo et al. 2017; Dötterl and Gershenzon 2023; Muhlemann et al. 2014). Thus far, among orchid species, the reported SCs involved in sexual deception span across all the major classes of VOCs i.e.: fatty acid derivatives—alkanes, alkenes, alcohols, carboxylic acids, esters, lactones; isoprenoids—monoterpenes, diterpenes, sesquiterpenes; benzenoids and phenylpropanoids (Perkins et al. 2023). In addition, the molecules involved in sexual deception ranged from long‐chain hydrocarbons, hydroxy acids, keto acids, to volatile esters and pyrazine derivatives. For example, alkanes and alkenes are known as components of the sex pheromone mimicry across bee‐ pollinated *Ophrys* (Table 1) (Perkins et al. 2023). In Australian sexually deceptive orchids, hydroxymethylpyrazines and a β‐hydroxylactone (drakolide) have been identified as pollinator attractants in *Drakaea sp*. (Bohman et al. 2014; Bohman et al. 2020b). In *Drakaea glyptodon*, pollinators are attracted with a blend of alkylpyrazines and hydroxymethylpyrazines (Bohman and Peakall 2014; Bohman et al. 2014). Moreover, acetophenones and monoterpenes have been identified as pollinator attractants in *Caladenia plicata* (Xu et al. 2017), and tetrahydrofuran acid derivatives in *Cryptostylis ovata* (Bohman et al. 2019). Not long ago, methylthiophenol compounds (Table 1) were added to the list. These phenolic compounds were not known as SCs in any other organisms, nevertheless, as they are perceived by thynnine wasps, they may represent an important class of SCs within the *Zaspilothynnus* and *Campylothynnus* and many other orchid‐pollinating wasps (Bohman et al. 2017; Bohman et al. 2018a). Outside of the Orchidaceae, the *Serapias* and the *Oncocyclus* irises (Iridaceae) attract their pollinators through sexual deception via pseudocopulation, with this evolutionary transition hypothesized to be influenced by the specific ratio of of *n*‐alkanes and *n*‐alkenes in their floral scent (Vereecken et al. 2012). A list of recently studied plant SCs published from 2013 to 2023 is shown in Table 1 and will be discussed in the following section.

**Table 1:** Examples of SCs used in sexual deception (2013–2023).

Compounds	Class	Plant species	Family	Pollinator	Reference
Fatty acid derivatives					
Alkane	n‐pentadecane	*Ophrys insectifera*	Orchidaceae	*Argogorytes fargeii*	(Bohman et al. (2020c))
Alkene	tricosatriene	*Pterostylis*	Orchidaceae	fungus gnats	(Hayashi et al. (2021))
Alcohol	nonan‐2‐ol	*Ceropegia gerrardii*	Apocynaceae	*Desmometopa spp*.	(Heiduk et al. (2023))
Ketone	2‐hydroxy‐6‐methylacetophenone	*Caladenia plicata*	Orchidaceae	*Zeleboria sp*.	(Bohman et al. (2018b))
Ketone	heptan‐2‐one	*Ceropegia gerrardii*	Apocynaceae	*Desmometopa spp*.	(Heiduk et al. (2023))
Ester	octyl acetate				
Ester	methyl (S)−2‐ (tetrahydrofuran‐2‐yl)acetate	*Cryptostylis ovata*	Orchidaceae	*Lissopimpla excelsa*	(Bohman et al. (2019))
Ester	ethyl (S)−2‐(tetrahydrofuran‐2‐yl)acetate	*Cryptostylis ovata*	Orchidaceae	*Lissopimpla excelsa*	(Bohman et al. (2019))
Carboxylic acid	(S)−2‐(tetrahydrofuran‐2‐yl) acetic acid	*Cryptostylis ovata*	Orchidaceae	*Lissopimpla excelsa*	(Bohman et al. (2019))
Lactone	(16S,9Z)−16‐ethyl hexadec‐9‐enolide	*Disa forficaria*	Orchidaceae	beetles	(Cohen et al. (2021))
Lactone	drakolide 1	*Drakaea livida*	Orchidaceae	*Zeleboria sp*.	(Bohman et al. (2020b); Bohman et al. (2022))
Pyrazines	alkylpyrazine	*Drakaea glyptodon*	Orchidaceae	*Zaspilothynnus trilobatus*	(Bohman and Peakall (2014); Bohman et al. (2014))
Pyrazines	hydroxymethylpyrazine	*Drakaea glyptodon*	Orchidaceae	*Zaspilothynnus trilobatus*	(Bohman and Peakall (2014); Bohman et al. (2014))
Terpenoids					
Monoterpene	(*S*)‐β‐citronellol	*Caladenia plicata*	Orchidaceae	*Zeleboria sp*.	(Bohman et al. (2018b))
Monoterpens	a‐terpineol	*Ceropegia gerrardii*	Apocynaceae	*Desmometopa spp*.	(Heiduk et al. (2023))
Monoterpene	geraniol	*Ceropegia gerrardii*	Apocynaceae	*Desmometopa spp*.	(Heiduk et al. (2023))
Diterpene	( + )‐copalol	*Cryptanthus burle‐marxii*	Bromeliaceae	*Eulaema nigrita*	(Milet‐Pinheiro et al. (2021))
Benzenoids					
Benzenoids	benzyl acetate	*Ceropegia gerrardii*	Apocynaceae	*Desmometopa spp*.	(Heiduk et al. (2023))
Benzenoids	2‐(methylthio) benzene‐1,4‐diol	*Drakaea livida*	Orchidaceae	*Zaspilothynnus nigripes*	(Weinstein et al. (2022))
Benzenoids	4‐hydroxy‐3‐(methylthio) benzaldehyde	*Drakaea livida*	Orchidaceae	*Zaspilothynnus nigripes*	(Weinstein et al. (2022))
Phenylpropanoids					
(Methylthio)phenol	2‐(methylthio) benzene‐1,4‐diol	*Caladenia crebra*	Orchidaceae	*Campylothynnus flavopictus*	(Bohman et al. (2018a))
(Methylthio)phenol	4‐hydroxy‐3‐(methylthio) benzaldehyde	*Caladenia crebra*	Orchidaceae	*Campylothynnus flavopictus*	(Bohman et al. (2018a))
(Methylthio)phenol	Sulphur‐containing phenols	*Caladenia crebra*	Orchidaceae	*Campylothynnus flavopictus*	(Bohman et al. (2017))

## 4 A Single Component or Rather a Bouquet

Chemical signals may be made up of multi‐component blends, requiring pattern recognition at the receiver site or they may represent single compound systems that afford specialist receptors. On the one hand, some flowers e.g. *Ophrys* produce complex species‐specific mixtures of more than 100 compounds (Ayasse et al. 2011). On the other hand, attraction can be achieved by combinations of only a few chemical compounds, as in the case of *Ophrys speculum* which attracts its pollinator, *Campsoscolia ciliata*, with a mix of just eight compounds (Ayasse et al. 2003). While the biosynthesis of a unique compound requires a special set of enzymes, the formation of a qualitatively and quantitatively well‐defined blend of more or less ubiquitous compounds of closely related chemical structures may simply require fine‐tuning of enzymes already expressed in that species (Ayasse et al. 2011).

A recent study compared floral VOCs of 9 different plant species that are predominantly visited by honey bees and bumblebees. While comprising different volatile bouquets, flowers of all species had one thing in common ‐ E‐β‐ocimene, a principal component for attracting both honey bees and bumblebees (Dekebo et al. 2022). Since many years, E‐β‐ocimene has been known to act as a brood pheromone of honey bees with high occurrence in floral scents (Dekebo et al. 2022). In addition, this study reported that *Ailanthus altissima* flowers contained monoterpenes β‐linalool (39.1%) and hotrienol (32.1%) as predominant compounds (Dekebo et al. 2022). Namely, linalool is produced in mandibular glands of solitary bees and bumblebees, and acts as a pheromone that causes the males to aggregate (Borg‐Karlson et al. 1996).

**Figure 2:**
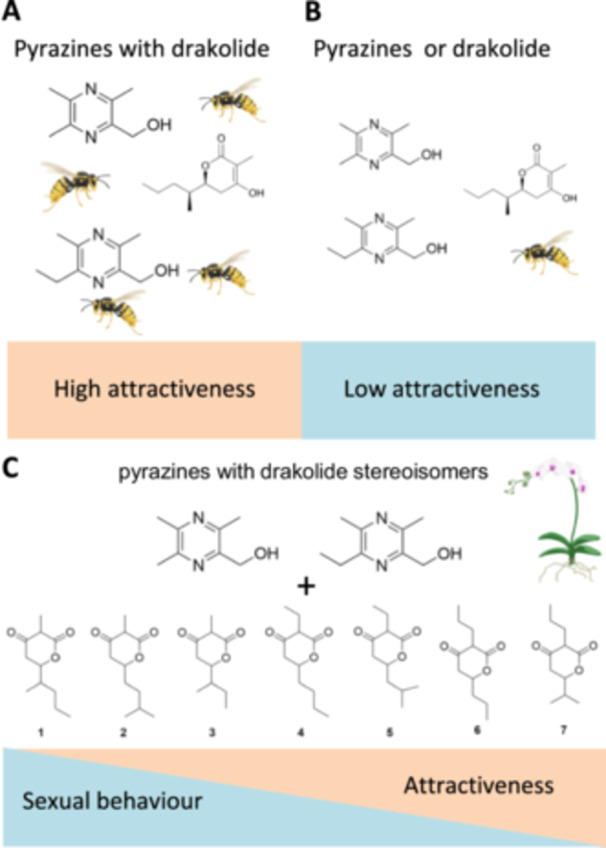
Sexual attraction of the male *Zeleboria sp*. pollinators by floral blends of a β‐ketolactone drakolide 1 and hydroxymethylpyrazines in *Drakea micrantha*. The blend is only attractive to the pollinating wasps when it contains both the drakolide 1 and a pyrazine (A), while the two compounds presented alone do not attract pollinators (B). Blends of pyrazines with drakolide stereoisomers showed dosage effect on sexual behavior and attractiveness (C). [Color figure can be viewed at wileyonlinelibrary.com]

In their recent work, Bohman and colleagues discovered that plants use blends of chemically unrelated compounds as sexual attractants (Bohman et al. 2022). Notably, to attract male *Zeleboria sp*. wasp pollinators, orchid *Drakaea micrantha* uses a blend of three compounds consisting of a β‐ketolactone (drakolide 1) and two specific hydroxymethylpyrazines 8 and 9. This floral bouquet was shown to be attractive only if required both a pyrazine and drakolide 1 (Figure 2A&B). Furthermore, experiments with drakolide stereoisomers featuring substituents at positions 3 and 6 resulted in reduced sexual behaviour and varying levels of attractiveness (Figure 2C). Interestingly, treatments with drakolides 2 and 3 with the same C‐3 methyl group as 1, elicited some activity, including pollinator landing and copulation. The activity further declined with drakolides 4 and 5, while the activity was minimal with the substitution of a propyl group at C‐3 in drakolides 6 and 7. Similarly, variation of the substituent on C‐6 also adversely affected attractiveness (Figure 2C) (Bohman et al. 2022). It remains unknown as to how two different biosynthetic pathways were co‐opted in the highly specific pollination of the sexually deceptive *Drakaea micrantha* and its pollinator. One hypothesis could be a general model for pollinator‐mediated speciation by Peakall and Whitehead, which assumes that gene duplication and/or allelic variation underpins pollinator switching (Peakall and Whitehead 2014). In this case, neutral mutations could diffuse through populations where they become available for the use and subsequent selection by a second pollinator (Bohman et al. 2022).

## 5 SCs as a Tool for Pollinator Conservation

**Figure 3:**
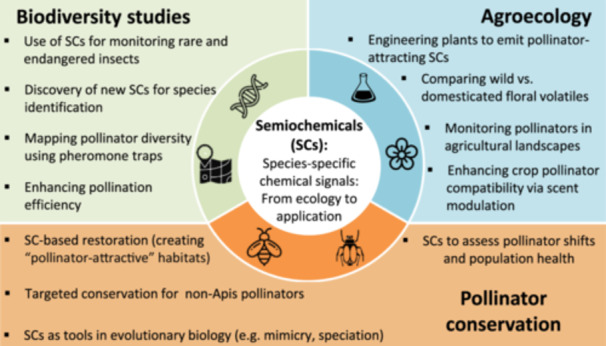
Applications of SCs in biodiversity research, agroecology and pollinator conservation. In **biodiversity research**, by mediating communication between organisms, SCs can aid in monitoring rare and endangered insect species and could be used for mapping pollinator hotspots and diversity patterns. Additionally, SCs can enhance pollination efficiency by influencing pollinator attraction and behavior. **Agroecology.** The use of SCs in pollinator research enables noninvasive assessment of pollinator health, distribution, and species diversity across habitats. Integrating SC‐based tools with conservation strategies enhances monitoring and preservation efforts in both natural and agricultural landscapes. **Pollinator conservation strategies.** Strategies aimed at pollinator conservation incorporate the development of pollinator‐supportive plant varieties including the conservation of non‐Apis pollinators and synthetic SCs. Furthermore, SCs are a powerful tool that could help address key questions about the evolution of behavior, speciation, adaptation, and coevolution. [Color figure can be viewed at wileyonlinelibrary.com]

What can we learn from SCs? SCs and specifically insect pheromones have a tremendous potential as monitoring tools in biodiversity and conservation research, particularly for endangered species, which has only been realized to a limited extent (Larsson 2016). Pheromone‐based trapping systems have been proven useful for monitoring rare and threatened saproxylic beetles and their predator (Larsson and Svensson 2009) and could also be implemented on rare pollinators (Figure 3, conservation monitoring). At the population level, SCs would provide an excellent means of monitoring population changes, identifying biodiversity hotspots and habitat thresholds for persistence of target species (Larsson 2016; Figure 3). Today, studies on SCs and pheromones in pest management prevail (Su et al. 2020; Liu et al. 2024), while only a few pheromones have been developed for biodiversity and conservation studies, including the identification and application of pheromones specifically for population monitoring (Larsson 2016). The attractiveness of many insect pheromones could facilitate monitoring at an unprecedented spatiotemporal resolution with great efficiency (Larsson 2016), and being sustainable and easily integrated, this approach would greatly aid in pollinator monitoring and conservation.

From the pollinator conservation perspective, studying *Ophrys sp*. is valuable having in mind the available genomic data and the fact that most *Ophrys sp*. produce hundreds of chemical compounds (Ayasse et al. 2011), and thus have the evolutionary potential to switch pollinators. Since most crops rely on a single pollinator ‐ the honey bee, it would be critical to identify and investigate diverse pollinator species for insect‐pollinated crops and determine which volatiles play key roles in attracting these alternative pollinators. Certainly, one of the important advantages of SCs is their high species specificity, often achieved by a combination of structurally similar molecules in a precise ratio, and related to their roles in mate communication and reproductive isolation. Last but not least, the biological efficacy and economic feasibility of pheromone production has also been recently demonstrated in plant factories by metabolic engineering (Wang et al. 2022).

One of the unanswered questions is whether the cultivated crops, which have undergone multiple domestication events and have been selected to improve yield, have also been altered in their volatile profiles and thus communication with pollinators. Notably, flower enlargement due to domestication has been reported to increase pollinator visitation rates (Chen et al. 2018; Sapir 2009), but how has this influenced plant‐pollinator coevolution requires further research. For example, a recent study compared the floral attributes and visitor interactions between sister taxa of domesticated and wild *Cucurbita* species, finding that the domesticated taxa had larger floral characteristics, higher pollen quantity, and a higher protein‐to‐lipid ratio, with *Eucera spp*. being most likely to visit all *Cucurbita* taxa (Glasser et al. 2023). Moreover, studies on highbush blueberry (*Vaccinium corymbosum* L.) have shown that crop domestication significantly alters the chemical composition of nectar and pollen, while reducing pollen chemical diversity in cultivated plants compared to wild ones, with potential negative implications for pollinator health via changes in pathogens. Another fascinating example of a direct influence of genetic variation on plant‐pollinator interactions has been reported in monkeyflowers (*Mimulus spp*.), in which variation in the *YELLOW UPPER (YUP)* gene caused changes in flower color patterns that in turn drive speciation with shifts in whether the flower is pollinated by bees or by hummingbirds (Liang et al. 2023). To date, the research on the effects of domestication on floral reward chemistry and plant‐pollinator interactions remains limited. To ensure long‐term sustainability in agroecosystems, one of the aims of plant breeding strategies should be to identify and prioritize the “pollinator‐friendly” genotypes for a variety of insect species (Figure 3, Pollinator conservation strategy).

## 6 Concluding Remarks and Future Perspectives

Deceptive pollination strategies showcase the intricate interplay between sensory exploitation and adaptive evolution. Yet, beyond their ecological intrigue, these strategies hold unexplored potential for conservation, agriculture and biotechnological applications. As pollinators face alarming declines, understanding plant‐pollinator interactions at the chemical level is no longer a purely academic pursuit—it is a necessity for ecosystem stability and food security.

Future research should prioritize the discovery of novel SCs and the elucidation of their ecological roles, particularly in shaping pollinator learning, adaptation, and plant speciation. One major direction involves identifying the genetic and metabolic pathways underlying SC production and understanding how small genetic changes can alter chemical signals and influence pollinator behavior. Investigating how pollinators perceive, process, and retain these cues—especially how they learn and remember them—will provide valuable insights into the effectiveness of plant deception and its ecological consequences. There is also a need to explore how deceptive strategies shape, and are shaped by, evolutionary pressures in plant‐pollinator relationships, particularly within complex ecosystems. Metabolomics and other omics technologies offer powerful tools to discover new signaling compounds and link them to ecological outcomes and adaptive benefits. On the applied side, research should investigate how synthetic versions of SCs might enhance pollination services in agriculture, particularly where natural interactions have been disrupted. Such tools could be used to guide pollinators more efficiently to crops or ecological restoration sites. Moreover, incorporating chemical signaling knowledge into conservation strategies may also improve habitat restoration by increasing floral attractiveness to key pollinators. Lastly, it is essential to understand how environmental factors—such as climate change, pollution, and land‐use shifts—impact both the emission and perception of SCs.

Beyond scientific inquiry, there is an urgent need for interdisciplinary collaboration. Chemists, geneticists, ecologists, and conservationists must work together to develop pheromone‐based conservation strategies, create pollinator‐friendly agroecosystems, and explore synthetic biology applications for pest and pollinator management. While multi‐omics technologies and high‐resolution imaging technologies have propelled our understanding of pollination strategies, integrating these fields with behavioral ecology and evolutionary biology will be pivotal to future progress. Finally, engineering crop species to produce specific SCs opens a virtually limitless realm of research, with the capacity to substantially enhance pollinator attraction and enable more sustainable pest management.

## Conflicts of Interest

The authors declare no conflicts of interest.

## Data Availability

Data sharing is not applicable to this article as no new data were created or analyzed in this study.
